# Host Defense Peptide LL-37-Mediated Chemoattractant Properties, but Not Anti-Inflammatory Cytokine IL-1RA Production, Is Selectively Controlled by Cdc42 Rho GTPase *via* G Protein-Coupled Receptors and JNK Mitogen-Activated Protein Kinase

**DOI:** 10.3389/fimmu.2018.01871

**Published:** 2018-08-13

**Authors:** Mahadevappa Hemshekhar, Ka-Yee Grace Choi, Neeloffer Mookherjee

**Affiliations:** ^1^Manitoba Centre for Proteomics and Systems Biology, Department of Internal Medicine, University of Manitoba, Winnipeg, MB, Canada; ^2^Department of Immunology, University of Manitoba, Winnipeg, MB, Canada

**Keywords:** host defense peptide, cathelicidin, LL-37, Cdc42 RhoGTPase, inflammation, JNK mitogen-activated protein kinase, cell migration

## Abstract

The human host defense peptide LL-37 promotes immune activation such as induction of chemokine production and recruitment of leukocytes. Conversely, LL-37 also mediates anti-inflammatory responses such as production of anti-inflammatory cytokines, e.g., IL-1RA, and the control of pro-inflammatory cytokines, e.g., TNF. The mechanisms regulating these disparate immunomodulatory functions of LL-37 are not completely understood. Rho GTPases are GTP-binding proteins that promote fundamental immune functions such as chemokine production and recruitment of leukocytes. However, recent studies have shown that distinct Rho proteins can both negatively and positively regulate inflammation. Therefore, we interrogated the role of Rho GTPases in LL-37-mediated immunomodulation. We demonstrate that LL-37-induced production of chemokines, e.g., GRO-α and IL-8 is largely dependent on Cdc42/Rac1 Rho GTPase, but independent of the Ras pathway. In contrast, LL-37-induced production of the anti-inflammatory cytokine IL-1RA is not dependent on either Cdc42/Rac1 RhoGTPase or Ras GTPase. Functional studies confirmed that LL-37-induced recruitment of leukocytes (monocytes and neutrophils) is also dependent on Cdc42/Rac1 RhoGTPase activity. We demonstrate that Cdc42/Rac1-dependent bioactivity of LL-37 involves G-protein-coupled receptors (GPCR) and JNK mitogen-activated protein kinase (MAPK) signaling, but not p38 or ERK MAPK signaling. We further show that LL-37 specifically enhances the activity of Cdc42 Rho GTPase, and that the knockdown of Cdc42 suppresses LL-37-induced production of chemokines without altering the peptide’s ability to induce IL-1RA. This is the first study to demonstrate the role of Rho GTPases in LL-37-mediated responses. We demonstrate that LL-37 facilitates chemokine production and leukocyte recruitment engaging Cdc42/Rac1 Rho GTPase *via* GPCR and the JNK MAPK pathway. In contrast, LL-37-mediated anti-inflammatory cytokine IL-1RA production is independent of either Rho or Ras GTPase. The results of this study suggest that Cdc42 Rho GTPase may be the molecular switch that controls the opposing functions of LL-37 in the process of inflammation.

## Introduction

The human host defense peptide LL-37 is a 37-amino acid cationic peptide with immunomodulatory functions required for resolution of infections and regulation of inflammation (1–5). LL-37 mediates both pro- and anti-inflammatory responses; LL-37 can promote chemokine production, recruitment of leukocytes, differentiation of macrophages and dendritic cells, polarization of T-cells and angiogenesis, all functions classically defined as pro-inflammatory (6–8). In contrast, LL-37 also exhibits anti-inflammatory properties such as intervening in toll-like receptor signaling to suppress endotoxin-induced pro-inflammatory cytokines, regulating signaling mechanisms and downstream responses induced in the presence of pro-inflammatory cytokines such as IL-32, and promoting the production of anti-inflammatory cytokines such as interleukin-1-receptor antagonist (IL-1RA) (4, 9, 10). Previous studies have shown that specific G protein-coupled receptors (GPCRs) are involved in the immunomodulatory activity of LL-37 *via* activation of the mitogen-activated protein kinase (MAPK) pathway, in particular, the p38 MAPK (8, 11). GPCRs are known to trigger complex signaling networks that involve Rho GTPases and MAPK pathways (12). Involvement of Rho GTPases in the immunomodulatory functions of LL-37 has not been fully elucidated.

Rho GTPases are guanine nucleotide-binding proteins (G proteins) belonging to the subgroup of the Ras superfamily, which includes proteins such as Rho, Rac, and Cdc42 (13). Activation of Rho GTPases is controlled by a set of proteins known as guanine nucleotide exchange factors (GEF), which facilitates the switch of inactive GDP-bound GTPase to the GTP-bound active form (14). Specific Rho proteins regulate cytoskeletal rearrangements by influencing the process of stress fiber formation (Rho), filopodial extensions (Cdc42), and lamellipodia and membrane ruffles (Rac) (13, 15). Consequently, these proteins play a major role in actin-mediated processes such as cell movement, polarity, shape, and axonal guidance, and processes critical in cell migration and metastatic behavior (16, 17). In addition, recent studies have demonstrated that Rho GTPases can both promote and control the process of inflammation (17, 18). For example, Langert et al. showed that Cdc42 Rho GTPase facilitate intracellular trafficking of the pro-inflammatory cytokine TNF-α, and release of TNF-α-mediated chemokine CCL2/monocyte chemotactic protein-1 (MCP-1), in peripheral nerve microvascular endothelial cells (19). Moreover, distinct Rho proteins can both negatively and positively regulate the activity of the transcription factor NF-κB to either control or enhance inflammation (20). Therefore, in this study, we interrogated the involvement of Rho GTPases in the opposing immune functions of the human host defense peptide LL-37, as this peptide facilitates both the control and promotion of inflammation (1–5, 10, 21–23).

In this study, we demonstrate that LL-37-mediated production of chemokines and consequent recruitment of leukocytes is largely dependent on Cdc42/Rac1 Rho GTPase, and that this process involves GPCRs and the JNK MAPK pathway. In contrast, LL-37-induced production of the anti-inflammatory cytokine IL-1RA is independent of Rho GTPases. We show that LL-37 increases Cdc42 Rho GTPase activity, and that the knockdown of Cdc42 selectively suppresses LL-37-mediated production of chemokines, but not that of IL-1RA. Overall, our results suggest that Cdc42 Rho GTPases may serve as the molecular switch to regulate the dichotomy between the pro- and anti-inflammatory functions of LL-37.

## Materials and Methods

### Reagents

Peptides LL-37 (LLGDFFRKSKEKIGKEFKRIVQRIKDFLRNLVPRTES) and a paired scrambled LL-37 peptide, sLL-37 (RSLEGTDRFPFVRLKNSRKLEFKDIKGIKREQFVKIL) were synthesized from CPC Scientific (Sunnyvale, CA, USA). The peptides were re-suspended in endotoxin-free water and stored at −20°C until needed. *E. coli* lipopolysaccharide (LPS) was obtained from Sigma-Aldrich (Oakville, ON, Canada). Pharmacological inhibitors ML141 was obtained from Millipore Ltd. (ON, Canada), farnesylthiosalicylic acid (FTS) from Cayman Chemicals Co. (MI, USA), and pertussis toxin (PTx) from Thermo fisher scientific (MA, USA). Human recombinant macrophage colony-stimulating factor (MCSF) was obtained from R&D Systems (Minneapolis, MN, USA). Antibodies specific for phosphorylated-MAPK; phospho-JNK (Thr183/Tyr185), phospho-p38 (Thr180/Tyr182) and phospho-Erk1/2 (Thr202/Tyr204), and antibodies specific for total JNK, p38, and Erk MAPK, were all purchased from Cell Signaling Technology (Denver, CO, USA).

### Cell Culture

Human monocytic THP-1 cells (ATCC^®^ TIB-202™) were cultured as previously described (4) in complete RPMI-1640 medium containing 1 mM sodium pyruvate and 10% (v/v) fetal bovine serum, at 37°C in a 5% CO_2_ humidified incubator. THP-1 cells (2 × 10^5^ cells/ml) were differentiated into plastic-adherent macrophage-like cells with 50 ng/ml of PMA (Sigma-Aldrich) and rested for an additional 24 h before treatment with various stimulants, as previously described (4, 10). Plastic-adherent macrophage-like THP-1 cells were treated with various inhibitors; either Cdc42 inhibitor ML141 (10 µM), Ras inhibitor FTS (10 µM each), or GPCR inhibitor PTx (100 ng/ml) as indicated, for 1 h prior to stimulation. Cells were stimulated with peptides either LL-37 or sLL-37 (5 µM each), LPS (10 ng/ml), or recombinant IL-32γ (20 ng/ml). Tissue culture (TC) supernatants were obtained after 24 or 48 h as indicated, centrifuged at 250 × *g* for 5 min to obtain cell-free samples, aliquoted, and stored at −20°C for further analysis. Cellular cytotoxicity was determined by monitoring the release of the enzyme lactate dehydrogenase (LDH) in the TC supernatants after 24 h of stimulation, using a colorimetric detection assay from Roche Diagnostic (Laval, QC, Canada) according to the manufacturer’s instructions.

### Human Peripheral Blood-Derived Mononuclear Cells (PBMC) Isolation

Venous blood was collected from healthy volunteers with written informed consent, according to a protocol approved by the University of Manitoba Research Ethics Board. Blood was diluted with complete RPMI-1640 medium (1:1) and fractionated over Ficoll-Paque Plus (GE Healthcare Life Sciences, Baie d’Urfe, QC, Canada). The buffy coat was isolated and washed twice in complete RPMI-1640 medium (300 × *g* for 10 min). PBMCs were isolated from the buffy coat as previously described (10). PBMCs were seeded (1 × 10^6^ cells/ml/well) into 24-well TC plates and rested for 2 h at 37°C in a humidified 5% CO_2_ incubator before stimulations.

### Human Monocyte-Derived Macrophages (MDM) Culture

3 × 10^6^ human PBMC/ml/well was seeded in complete RPMI-1640 medium into 24-well TC plates, and 2 ml of 5 × 10^6^ PBMC/ml/well was seeded into 6-well TC plates. The cells were incubated for 16 h at 37°C in a humidified 5% CO_2_ incubator. Non-adherent cells were removed and the adherent cells were differentiated into MDM in complete RPMI-1640 medium supplemented with 50 ng/ml of recombinant human MCSF for additional 6 days as previously described by us (10). Briefly, half the volume of the TC culture medium per well was replaced with fresh complete RPMI-1640 containing MCSF (50 ng/ml) every 48 h. On the day of stimulation, the culture medium was replaced with complete RPMI-1640 and the MDM were rested for 1 h at 37°C in a humidified 5% CO_2_ incubator before various stimulations.

### Human Neutrophil Isolation

Venous blood was collected from healthy volunteers in EDTA vacutainer tubes with written informed consent, according to a protocol approved by the University of Manitoba Research Ethics Board. Human neutrophils were isolated using EasySep™ Direct Human Neutrophil Isolation Kit (STEMCELL technologies Canada Inc., Vancouver, BC, Canada) according to the manufacturer’s protocol. Briefly, ~25 ml of blood was mixed gently with the isolation cocktail and 50 µl of RapidSpheres™ provided in the kit, and incubated for 5 min at room temperature. DPBS (containing 1 mM EDTA and free of Ca^2+^ and Mg^2+^) was used to make up the total volume to 50 ml, mixed gently followed by magnetic separation for 10 min. The enriched clear cell suspension was subjected to magnetic separation using RapidSpheres™, according to the manufacturer’s instructions, to obtain enriched human neutrophils.

### Cdc42 and Rac1 Activation G-LISA Assays

The Cdc42 and Rac1 G-LISA^®^ kits (Cytoskeleton, Inc., Denver, CO, USA) were used to monitor the activation of these specific Rho GTPases, according to the manufacturer’s instructions. Briefly, plastic-adherent macrophage-like THP-1 cells were serum starved for 1 h (except this assay, all other experiments were performed in the presence of serum). Subsequently, the cells were stimulated with either LL-37 or sLL-37 (5 µM each) or LPS (10 ng/ml) for 5 min (the time point was selected based on optimization studies monitoring the kinetics of response from 5 to 60 min). The cells were washed with cold PBS and lysed using the lysis buffer provided with the kit. The protein concentration in the lysates was determined using a micro-bicinchoninic acid (micro BCA) assay (Thermo Scientific, IL, USA). Cell lysates were aliquoted and stored at −80°C until used. Activation of Cdc42 and Rac1 GTPases were monitored in the cell lysates (25 µg protein), using the respective colorimetric G-LISA assays according to the manufacturer’s instructions. Specific activated GTPases provided in the kits were used as positive controls for the assays.

### Cdc42 SiRNA Knockdown

Cdc42 Accell SiRNA smartpool and a non-target Accell SiRNA smartpool were obtained from Dharmacon™ (Lafayette, CO, USA). Human monocytic THP-1 cells (3 × 10^6^ cells) were suspended in 5 ml Accell siRNA delivery media (Dharmacon™) treated with either human Cdc42 Accell SiRNA smartpool (1 µM) or non-target Accell SiRNA smartpool (1 µM). Wild-type untreated cells (WT) or cells treated with 50 µl SiRNA buffer were used as paired controls. Wild-type cells (WT) and cells treated with either Cdc42 Accell SiRNA smartpool (KD), non-target control Accell SiRNA smartpool (NTC), or siRNA delivery buffer (BC) were incubated at 37°C in a humidified 5% CO_2_ incubator for 96 h. Subsequently, the cells were differentiated into plastic-adherent macrophage-like cells as described above. Cells were stimulated with peptides either LL-37 or sLL-37 (5 µM each), or LPS (10 ng/ml). Production of chemokines GRO-α and IL-8, and cytokine IL-1RA were monitored by ELISA in TC supernatants after 24 and 48 h, respectively.

### ELISA

Production of cytokine IL-1RA, and chemokines GRO-α and IL-8 were monitored in the TC supernatants using antibody pairs from R&D Systems, and MCP-1 using antibody pair from eBiosciences (Thermo Fisher Scientific), as per the manufacturers’ instructions. Serial dilutions of the recombinant cytokines or chemokines were used to establish a standard curve for evaluation of the cytokine/chemokine concentrations in the TC supernatants (10).

### PathScan^®^ Sandwich ELISA for Quantitative Assessment of JNK MAPK Phosphorylation

Plastic-adherent macrophage-like THP-1 cells in 60-mm TC plates, and human MDMs in 6-well TC plates, were preincubated with Cdc42 inhibitor ML141 (10 µM) for 1 h prior to stimulation with peptides either LL-37 or sLL-37 (5 µM each), or IL-32 (20 ng/ml). As we have previously shown that the cytokine IL-32γ (20 ng/ml) increases the phosphorylation of JNK MAPK (10), recombinant IL-32 was used as a positive control. Cells were washed with PBS and lysed on the plate with 400 µl of lysis buffer containing 1 mM phenylmethylsulfonyl fluoride provided in the PathScan^®^ phospho-JNK (Thr183/Tyr185) ELISA kit (Cell Signaling Technology). Total protein concentration was quantified using a micro-BCA assay. Cell lysates (300 µg/ml of total protein) were incubated with phospho-JNK (Thr183/Tyr185) rabbit mAb-coated plates in the ELISA kits, which captures the phospho-proteins. A total JNK (L7E7) mouse mAb and horseradish peroxidase (HRP)-linked anti-mouse IgG provided in the kit were used to detect the captured proteins. Tetramethylbenzidine was used as a substrate for HRP, and the magnitude of color intensity was used to quantify phosphorylation of JNK proteins, as the quantity of phospho-JNK (Thr183/Tyr185) protein is proportional to the magnitude of light emission upon addition of chemiluminescent reagent.

### Western Blots

Plastic-adherent macrophage-like THP-1 cells were pre-incubated with ML141 (10 µM) for 1 h prior to stimulation with peptides either LL-37 or sLL-37 (5 µM each), recombinant IL-32γ (20 ng/ml), for 15 min. Cells were washed with cold PBS and scraped using a cell scraper, and further washed with cold PBS to obtain cell pellets. Cells were lysed in lysis buffer [20 mM Tris–HCl pH 7.5, 150 mM NaCl, 1 mM EDTA, 1 mM EGTA, 1 mM sodium fluoride, 1 mM sodium orthovanadate, 25 mM sodium pyrophosphate, protease inhibitor cocktail (Sigma-Aldrich), and 1% (v/v) Triton X-100]. Total protein concentration in the cell lysate was determined using a micro BCA assay (Thermo Scientific, Rockford, IL, USA). Equal amounts of protein (20 µg) were resolved on 4–12% NuPAGE Bis-Tris gels (Invitrogen Corporation, Burlington, ON, Canada), followed by transfer to nitrocellulose membranes (Millipore, MA, USA). Membranes were blocked with TBST (20 mM Tris–HCl pH 75, 150 mM NaCl, 0.1% Tween-20) containing 3% (w/v) BSA. Phosphorylation of JNK (Thr183/Tyr185), p38 (Thr180/Tyr182), and Erk1/2 (Thr202/Tyr204) MAPK was determined using anti-human phospho-site-specific antibodies (Cell Signaling Technology). Antibodies to paired total JNK, p38, and Erk1/2 MAPK (Cell Signaling Technology) were used for comparative densitometry analyses, and antibody to β-actin (Cell Signaling Technology) was used to normalize protein loading. The membranes were probed with the various antibodies in TBST containing 1% (w/v) BSA. Affinity purified HRP-linked secondary antibodies were used for detection, and the membranes were developed with an Amersham ECL detection system (GE Healthcare) according to the manufacturer’s instructions.

### RNA Isolation and Quantitative Real-Time PCR (qRT-PCR)

Plastic-adherent macrophage-like THP-1 cells were lysed and total RNA isolated using the Qiagen RNeasy Plus mini kit according to the manufacturer’s instructions. Total RNA was eluted in RNase-free water (Ambion). RNA concentration and purity were assessed using a NanoDrop 2000 Spectrophotometer (ThermoFisher Scientific). mRNA expression was analyzed using a SuperScript III Platinum Two-Step qRT-PCR kit with SYBR Green (Invitrogen, Burlington, ON, Canada) according to the manufacturer’s instructions, using the ABI PRISM 7300 Real-Time PCR System (Applied Biosystems, Foster City, CA, USA). Briefly, 800 ng of total RNA was reverse transcribed in a 20 µl reaction volume for 10 min at 25°C, followed by 50 min at 42°C, and the reaction was terminated by incubating the reaction mixture at 85°C for 5 min. cDNA was aliquoted and stored at −20°C until used. For qRT-PCR amplification, a reaction mix (12.5 µl) containing 2.5 µl of 1/10 diluted cDNA template, 6.25 µl of Platinum SyBr Green qPCR-Super-Mix UDG with Rox reference, 0.5 µl of 10 µM primer mix, and 3.25 µl of RNase-free water were used. The primers used for qRT-PCR are listed in Table 1. Product specificity was determined by melting curve analysis. Fold changes of mRNA expression were calculated after normalization to 18s rRNA, and using the comparative Ct method (24).

**Table 1:** List of primers used for quantitative real-time PCR.

Gene	Forward primer (5′–3′)	Reverse primer (5′–3′)
GRO-α	TCCTGCATCCCCCATAGTTA	CTTCAGGAACAGCCACCAGT
IL-8	AGACAGCAGAGCACACAAGC	AGGAAGGCTGCCAAGAGAG
18s rRNA	GTAACCCGTTGAACCCCATT	CCATCCAATCGGTAGTAGCG

### Cell Migration Assays

Human PBMCs were treated with Cdc42/Rac1 inhibitor ML141 (10 µM) for 1 h prior to stimulation with either LL-37 (5 µM) or sLL-37 (5 µM). TC supernatants were collected after 24 h and used in the bottom chamber of Transwell plates (Costar, Corning, NY, USA), to monitor the migration of either monocytic cells or neutrophils. Human THP-1 monocytes (3 × 10^5^ cells/well) or human neutrophils (6 × 10^5^ cells/well), in 200 µl RPMI 1640 medium supplemented with 10% FBS, were added to the upper chamber of the inserts of Transwell plates. An insert pore size of 5 µm was used for THP-1 cells, and 3 µm for human neutrophils as previously described (25, 26), and incubated at 37°C in a humidified chamber with 5% of CO_2_ for 30 min. TC supernatants (600 µl) collected from stimulated human PBMC (described above) were added to the bottom chamber of the Transwell plates, and further incubated for an additional 2 h. The number of cells that migrated to the bottom chamber was counted using a Scepter™ 2.0 Handheld Automated Cell Counter, Millipore Ltd. (ON, Canada). Human recombinant chemokines MCP-1, IL-8, and GRO-α (30 ng/ml each) were used in the bottom chamber in the migration assays as positive controls.

### Guanine Nucleotide Exchange Factor (GEF) Assay

Guanine nucleotide exchange factor assay was performed according to the manufacturer’s protocol provided by Cytoskeleton, Inc. The mixture containing Cdc42 RhoGTPases and fluorescent nucleotide analog *N*-methylanthraniloyl-GTP (mant-GTP) was added with test samples in a 384-well plate. The uptake of mant-GTP is measured using a fluorospectrometer at excitation 360 nm and emission 440 nm considering spectroscopic difference between free and GTPase-bound mant-GTP. Increase in the mant-GTP fluorescent intensity in the presence of a Cdc42 Rho GTPase indicates nucleotide uptake (or exchange for already bound nucleotide) by the GTPase (27).

### Statistical Analyses

GraphPad Prism 6 software was used for statistical analyses. One-way analysis of variance (ANOVA) followed by Bonferroni’s *post hoc* test was used for all assays using THP-1 cells. Kruskal–Wallis ANOVA followed by Dunn’s multiple comparison test was used for all experiments using human PBMC and MDM. A *p*-value of <0.05 was considered to be statistically significant.

## Results

### LL-37-Induced Chemokine Production Is Dependent on Cdc42/Rac1 Rho GTPase

**Figure 1:**
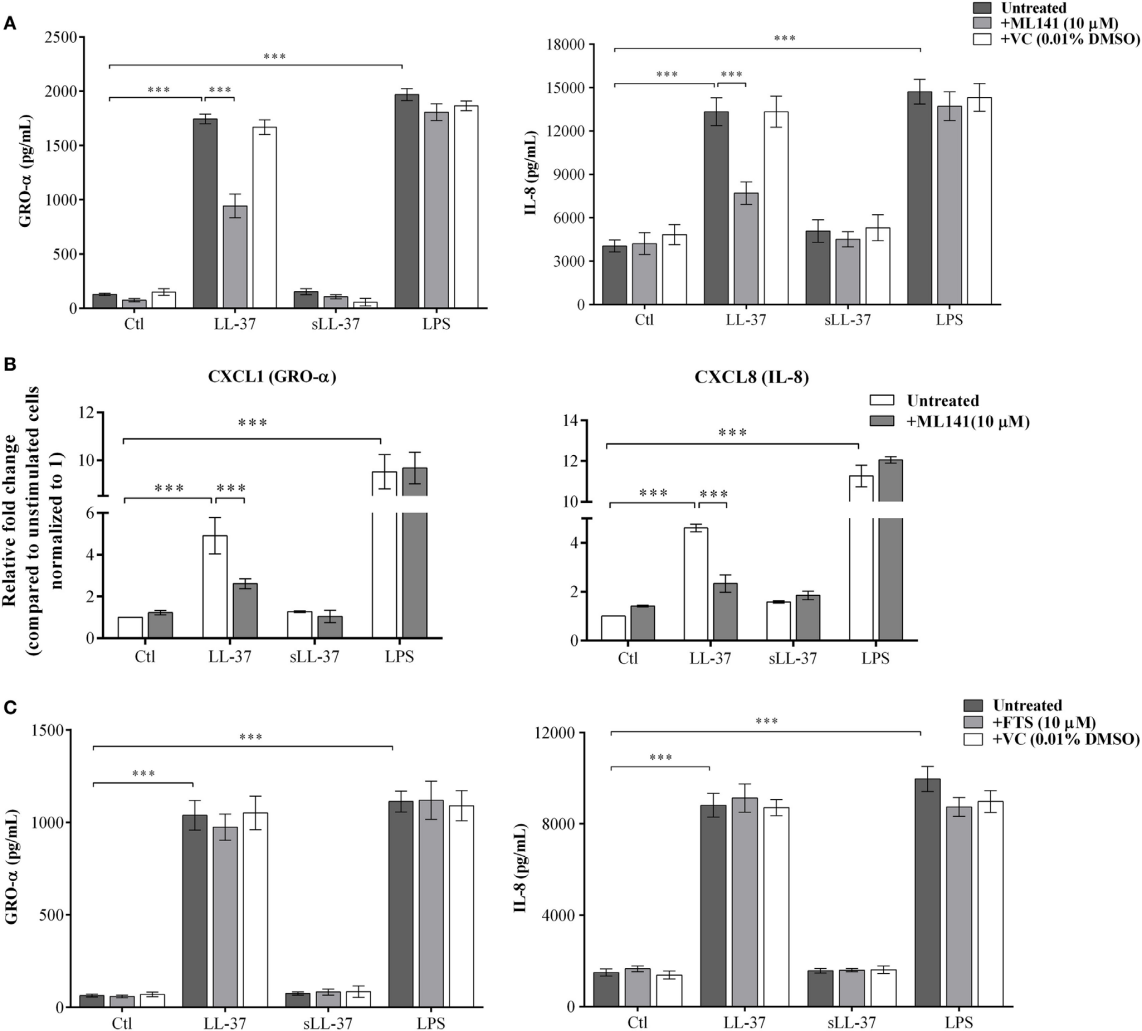
Cdc42/Rac1 GTPase controls LL-37-induced chemokine secretion. Macrophage-like THP-1 cells were pre-incubated with Cdc42/Rac1 inhibitor ML141 (10 µM) for 1 h, prior to stimulation with either LL-37 or sLL-37 (5 µM each), lipopolysaccharide (LPS) (10 ng/ml), or 0.01% DMSO as vehicle control (VC). **(A)** Tissue culture supernatants were monitored by ELISA for the production of chemokines GRO-α and IL-8 after 24 h. Results shown as mean ± SE of eight independent experiments (*n* = 8). **(B)** mRNA expressions were evaluated by quantitative real-time PCR for chemokines (*GRO-*α and *IL-8*) and anti-inflammatory cytokine *IL-1RA*, after 4 h. Relative fold changes were calculated compared to the expression in unstimulated cells, using the ΔΔCt method, after normalization with 18sRNA expression. Results shown as mean ± SE of three independent experiments. **(C)** Macrophage-like THP-1 cells were pre-incubated with the Ras inhibitor FTS (10 µM) for 1 h, prior to stimulation with either LL-37 or sLL-37 (5 µM each), or LPS (10 ng/ml). TC supernatants were monitored by ELISA for the production of chemokines GRO-α and IL-8 after 24 h. Results shown as mean ± SE of eight independent experiments (*n* = 8). Analysis of variance with Bonferroni’s *post hoc* test was used for statistical analyses (***p < 0.0005).

LL-37 is known to induce chemokine production in various cell types (4, 8, 10). Therefore, we examined the production of LL-37-induced chemokines GRO-α, IL-8, and MCP-1, in the presence and absence of specific pharmacological inhibitors. Macrophage-like THP-1 cells were pre-incubated with either Cdc42/Rac1 pharmacological inhibitor ML141 or Ras inhibitor FTS (10 µM each), for 1 h prior to stimulation with peptides either LL-37 (5 µM) or sLL-37 (5 µM), or bacterial LPS (10 ng/ml). TC supernatants were monitored by ELISA for the production of chemokines after 24 h. LL-37-induced production of GRO-α and IL-8 was significantly inhibited by ~50% in the presence of inhibitor ML141 (Figure 1A). LPS-induced chemokine production was not inhibited in the presence of ML141 (Figure 1A). MCP-1 was induced at very low levels, i.e., less than 10 pg/ml (data not shown), which is considered below the range of detection of the assay; therefore, we did not further use MCP-1 as a read out in THP-1 cells. Consistent with protein production (Figure 1A), LL-37-induced mRNA expression of chemokines (*GRO-*α and *IL-8*) was also suppressed by >3-fold, in the presence of ML141 (Figure 1B). In contrast, the Ras inhibitor FTS did not suppress either LL-37 or LPS-induced chemokine production (Figure 1C). These results demonstrated that LL-37-induced chemokine mRNA expression and protein production was dependent on Cdc42/Rac1 Rho GTPase, but independent of Ras GTPase.

### LL-37-Induced Anti-Inflammatory IL-1RA Production Is Independent of Rho GTPases

**Figure 2:**
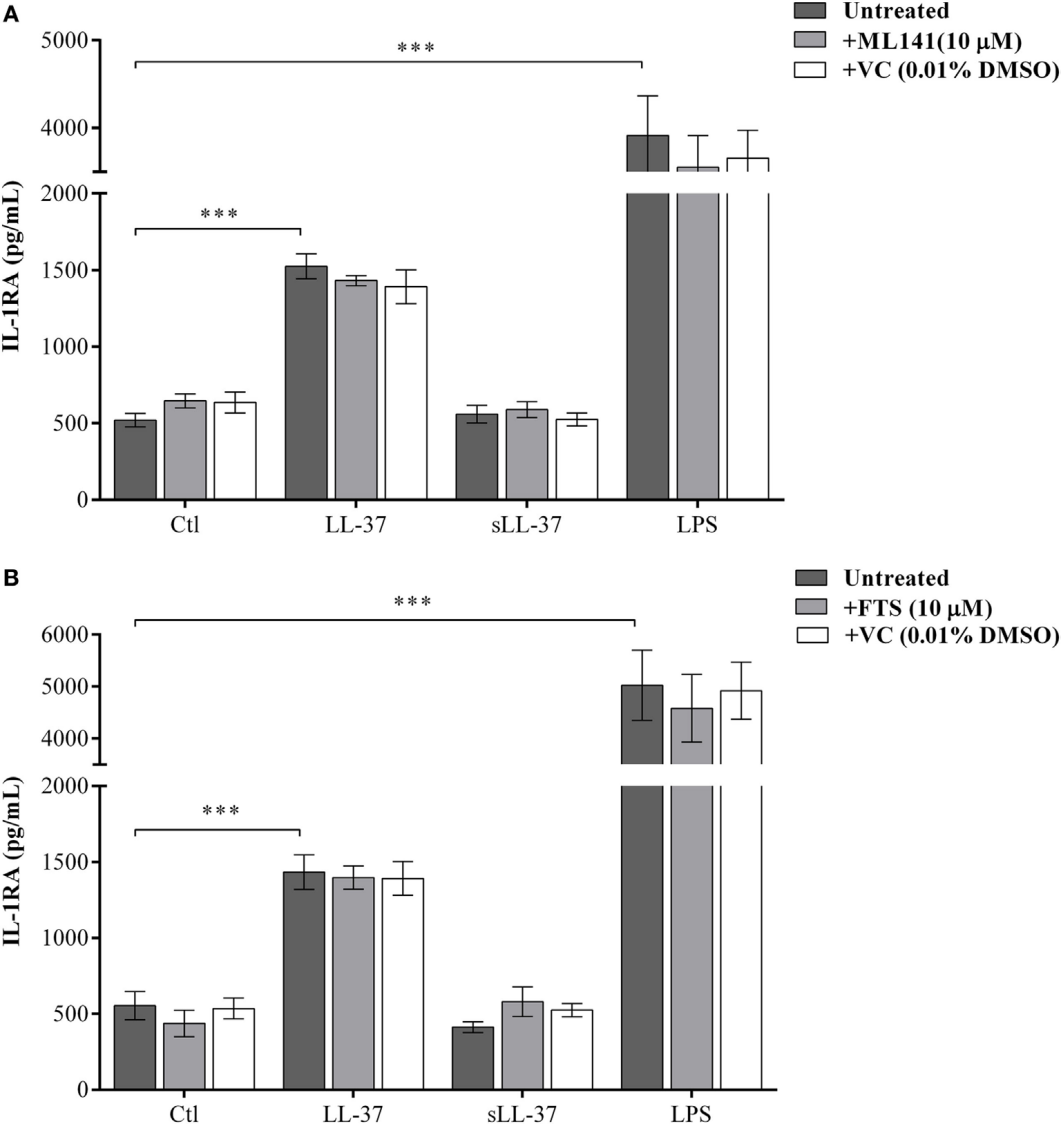
LL-37-induced anti-inflammatory cytokine IL-1RA production is independent of Rho and Ras GTPase activity. Macrophage-like THP-1 cells were pre-incubated with either **(A)** the Cdc42/Rac1 inhibitor ML141 (10 µM) or **(B)** Ras inhibitor FTS (10 µM), for 1 h prior to stimulation with either LL-37 (5 µM), sLL-37 (5 µM), lipopolysaccharide (10 ng/ml), or 0.01% DMSO as vehicle control (VC). Tissue culture supernatants were monitored by ELISA for the production of the anti-inflammatory cytokine IL-1RA after 48 h. Results shown as mean ± SE of eight independent experiments (*n* = 8). Analysis of variance with Bonferroni’s *post hoc* test was used for statistical analyses (***p < 0.0005).

We have previously shown that LL-37 induces the production of the anti-inflammatory cytokine IL-1RA in macrophage-like THP-1 cells and human PBMC (10). Therefore, we monitored the production of LL-37-induced IL-1RA, in the presence and absence of the pharmacological inhibitors either ML141 or FTS (10 µM each), in macrophage-like THP-1 cells. Production of IL-1RA was monitored in the TC supernatant by ELISA after 48 h based on our previous study (10). LL-37-induced production of IL-1RA was not inhibited by either ML141 (Figure 2A) or FTS (Figure 2B). These results demonstrated that the LL-37-induced production of IL-1RA was independent of both Cdc42/Rac1 and Ras GTPase pathways. Taken together, our results showed that LL-37-induced chemokine production, but not the anti-inflammatory cytokine IL-1RA, was dependent on the Cdc42/Rac1 Rho GTPase pathway. Moreover, LL-37-induced chemokine and IL-1RA production were both independent of Ras GTPase. Therefore, in further studies, we focused on the involvement of Cdc42/Rac1 Rho GTPase activity in LL-37-mediated responses.

### LL-37-Mediated JNK MAPK Phosphorylation Is Dependent on Cdc42/Rac1 Rho GTPase

**Figure 3:**
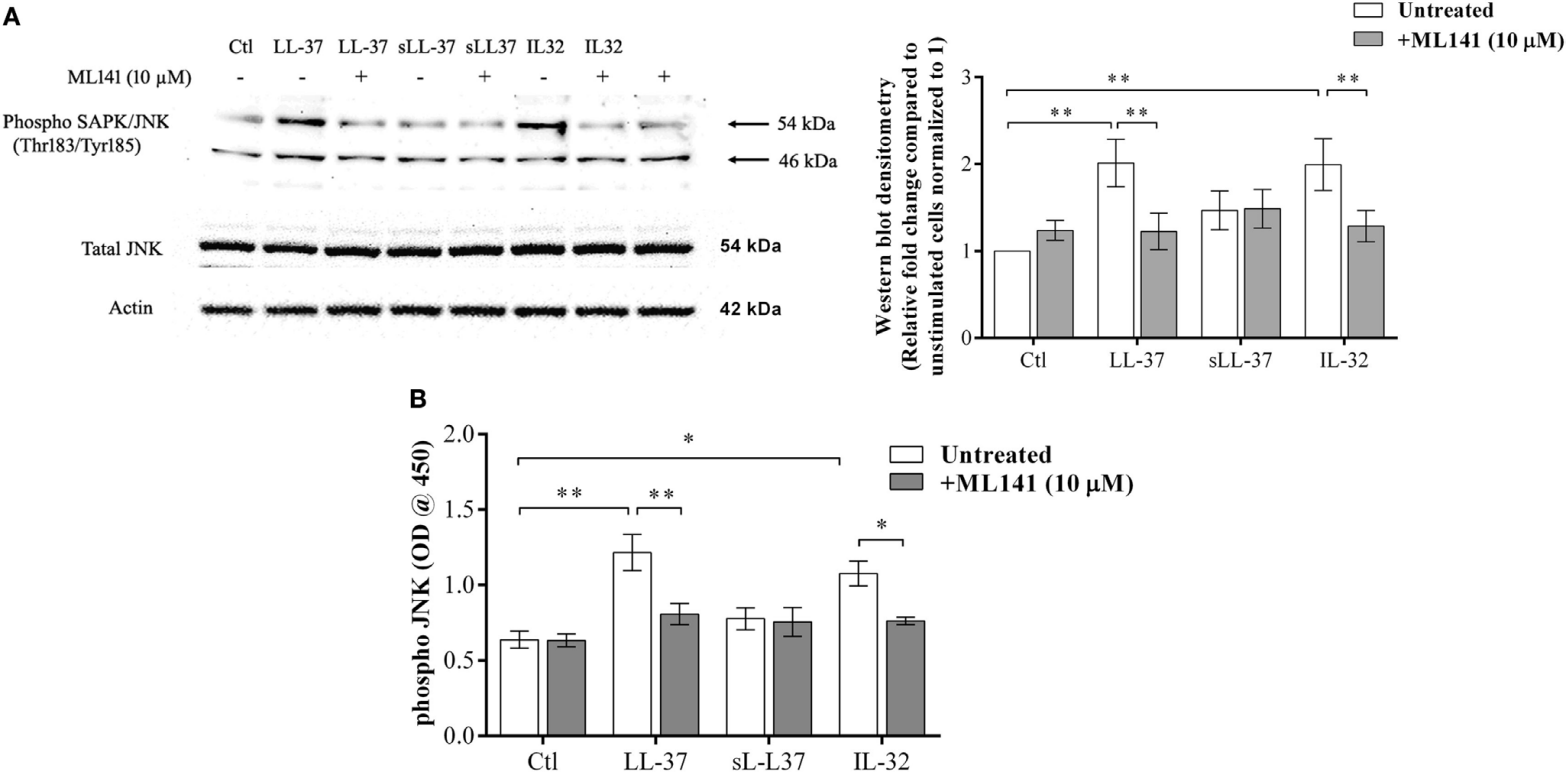
LL-37-mediated JNK mitogen-activated protein kinase (MAPK) phosphorylation is dependent on Cdc42/Rac1 RhoGTPase. Macrophage-like THP-1 cells were pre-incubated with Cdc42/Rac1 inhibitor ML141 (10 µM) for 1 h prior to stimulation with either LL-37 (5 µM), sLL-37 (5 µM), or recombinant human IL-32γ (20 ng/ml), for 15 min. **(A)** Cell lysates (each containing 20 µg total protein each) were probed with phospho-JNK (T183/Y185) antibody by western blots. Antibodies against total-JNK and β-actin were used as loading controls. Densitometry analyses for western blots represent relative fold change compared to unstimulated cells normalized to 1. Results shown as mean ± SE of five independent experiments (*n* = 5). **(B)** Cell lysates (40 µg each) were monitored by PathScan^®^ phospho-JNK (Thr183/Tyr185) Sandwich ELISA for relative quantitative estimation of JNK MAPK phosphorylation. Results shown as mean ± SE of four independent experiments (*n* = 4). Analysis of variance with Bonferroni’s *post hoc* test was used for statistical analyses (*p < 0.05, **p < 0.005).

Previous studies have demonstrated that LL-37-induced bioactivity involves MAPK signaling, in particular, the p38 and ERK1/2 MAPK pathway (10, 11). MAPK activity is also involved in signaling cascades triggered by Rho GTPases (12). Taken together, these studies suggest it is likely that the LL-37-Rho GTPase signaling axis would engage MAPK activity. Therefore, we further examined p38, Erk1/2, and JNK MAPK phosphorylation in response to LL-37, in the presence and absence of the Cdc42/Rac1 inhibitor ML141. IL-32γ was used as a control in this assay, as we have previously demonstrated that the phosphorylation of MAPKs, including JNK MAPK, is increased in response to IL-32γ in THP-1 cells (10). Western blot analyses demonstrated that LL-37-mediated phosphorylation of JNK MAPK was significantly inhibited in the presence of ML141 (Figure 3A). However, ML141 did not suppress LL-37-mediated phosphorylation of either p38 or Erk1/2 MAPK (Figure S1 in Supplementary Material). To provide a second line of evidence, we further used the PathScan^®^ ELISA assay to quantitatively estimate LL-37-mediated JNK MAPK phosphorylation in the presence and absence of the inhibitor ML141. Both densitometry assessment of western blots and PathScan^®^ ELISA demonstrated that LL-37-mediated JNK MAPK phosphorylation was significantly inhibited by ~50% in the presence of the Cdc42/Rac1 Rho GTPase inhibitor ML141 (Figures 3A,B, respectively). This was consistent with our results demonstrating that LL-37-induced chemokine production was suppressed by ~50% in the presence of ML141 inhibitor in THP-1 macrophage-like cells (Figure 1).

### LL-37-Induced Chemokine Production and JNK MAPK Phosphorylation Is Dependent on Cdc42/Rac1 Rho GTPase in Primary Human Blood-Derived Cells

**Figure 4:**
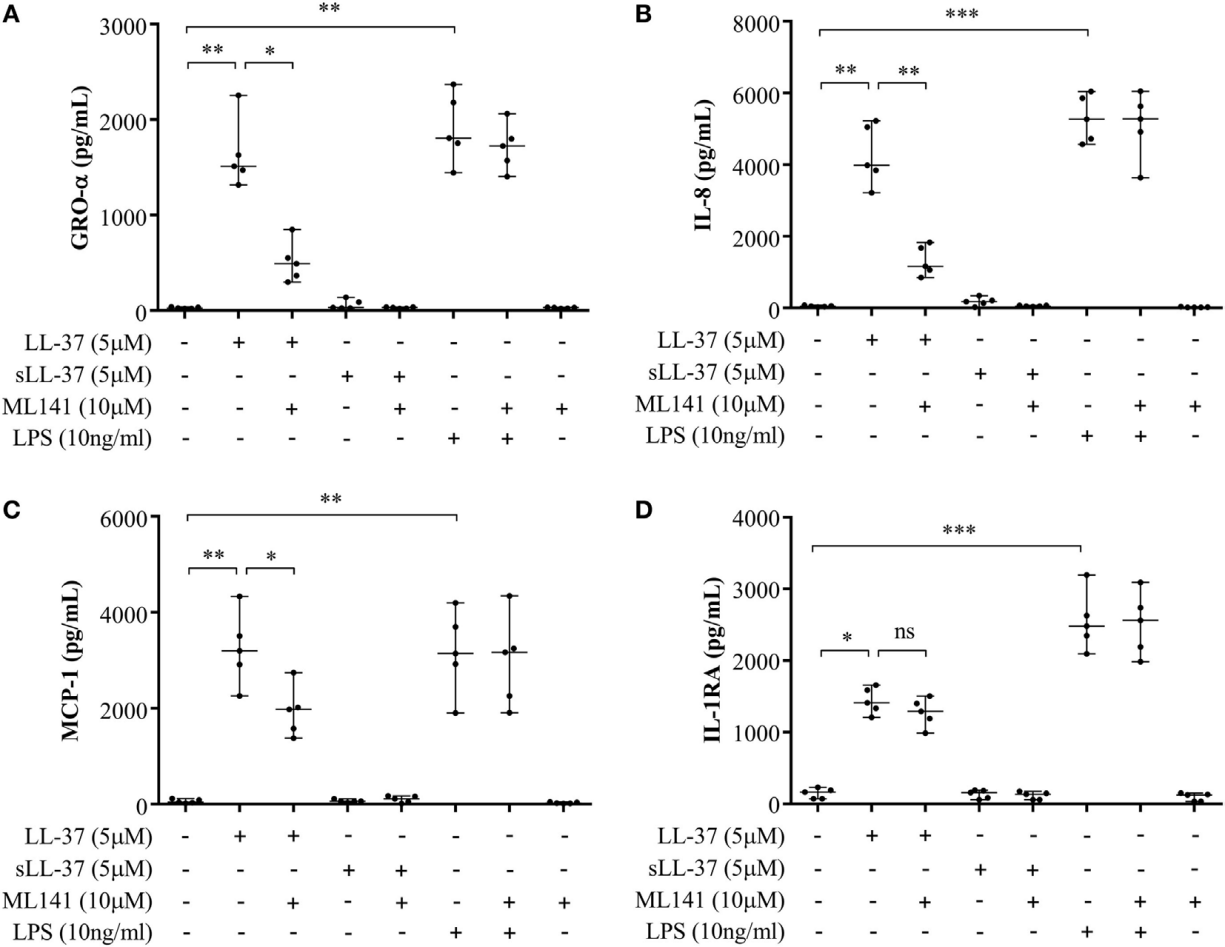
Cdc42/Rac1 Rho GTPase controls LL-37-induced chemokine secretion in human peripheral blood-derived mononuclear cells (PBMCs). Human PBMCs were pre-incubated with Cdc42/Rac1 inhibitor ML141 (10 µM) for 1 h prior to stimulation with either LL-37 (5 µM), sLL-37 (5 µM), or lipopolysaccharide (10 ng/ml). Tissue culture supernatants were monitored by ELISA for the production of chemokines **(A)** GRO-α, **(B)** IL-8, and **(C)** monocyte chemotactic protein-1 after 24 h, and **(D)** the anti-inflammatory cytokine IL-1RA after 48 h. Results shown are from five independent experiments (*n* = 5) using PBMCs isolated from independent donors, each dot represents an independent donor and the line shown is the median for each condition. Kruskal–Wallis analysis of variance with *Dunn’s* multiple comparison test was used for statistical analyses (*p < 0.05, **p < 0.005, ***p < 0.0005).

**Figure 5:**
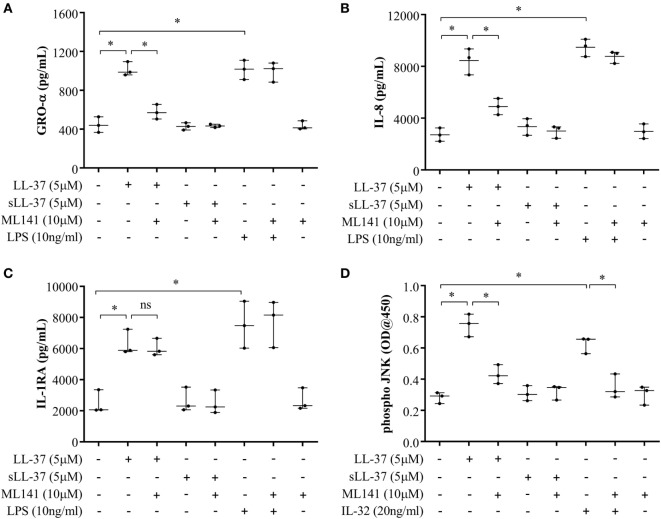
Cdc42/Rac1 Rho GTPase controls LL-37-induced chemokine secretion and JNK mitogen-activated protein kinase (MAPK) phosphorylation in human monocyte-derived macrophages (MDM). Human MDMs were pre-incubated with Cdc42/Rac1 inhibitor ML141 (10 µM) for 1 h prior to stimulation with either LL-37 (5 µM), sLL-37 (5 µM), or lipopolysaccharide (10 ng/ml). Tissue culture supernatants were monitored by ELISA for the production of chemokines **(A)** GRO-α and **(B)** IL-8 after 24 h, and **(C)** IL-1RA after 48 h. **(D)** Human MDMs were pre-incubated with Cdc42/Rac1 inhibitor ML141 (10 µM) for 1 h prior to stimulation with either LL-37 (5 µM), sLL-37 (5 µM), or recombinant human IL-32γ (20 ng/ml), for 15 min. Cell lysates (40 µg each) were monitored by PathScan^®^ phospho-JNK (Thr183/Tyr185) Sandwich ELISA for relative quantitative estimation of JNK MAPK phosphorylation. Results shown are from three independent experiments (*n* = 3) using MDMs isolated from independent donors. Each dot represents an independent donor and the line shown is the median for each condition. Kruskal–Wallis analysis of variance with *Dunn’s* multiple comparison test was used for statistical analyses (*p < 0.05).

Previous studies have shown that LL-37-mediated bioactivity in THP-1 macrophage-like cells, including induction of cytokines, is similar to that in primary human macrophages and PBMC (4, 10, 28, 29). Therefore, we further examined LL-37-mediated responses in human PBMC and MDM to validate our observations using macrophage-like THP-1 cells (Figures 1–3) in primary human cells. Human PBMC and MDM were stimulated with peptides LL-37 or sLL-37 (5 µM), in the presence or absence of Cdc42/Rac1 Rho GTPase inhibitor ML141. TC supernatants were monitored by ELISA for the production of chemokines (GRO-α, IL-8, and MCP-1) and the anti-inflammatory cytokine IL-1RA. LL-37-induced GRO-α, IL-8, and MCP-1 production were all significantly suppressed in the presence of the inhibitor ML141, in human PBMC (Figures 4A–C, respectively). Whereas, the inhibitor ML141 did not alter LL-37-induced IL-1RA production in human PBMC (Figure 4D). Similarly, LL-37-induced chemokines GRO-α and IL-8 were significantly suppressed in the presence of the inhibitor ML141 (Figures 5A,B, respectively), whereas IL-1RA production was not altered (Figure 5C), in human MDM.

We further examined LL-37-mediated JNK MAPK phosphorylation in the presence and absence of ML141 in human MDM. Consistent with the data in macrophage-like THP-1 cells (Figure 3), LL-37-mediated JNK MAPK phosphorylation was significantly suppressed by ~50% in the presence of inhibitor ML141, in human MDM (Figure 5D). Taken together, our results demonstrated that LL-37-induced chemokine responses, but not IL-1RA production, were dependent on the activity of Cdc42/Rac1 Rho GTPase in macrophage-like THP-1 cell line and in primary human cells such as PBMC and MDM. Moreover, LL-37-mediated JNK MAPK phosphorylation was also dependent on Cdc42/Rac 1 Rho GTPases in both macrophage-like THP-1 cell line and in primary human MDM.

### LL-37-Mediated Leukocyte Migration Is Dependent on Cdc42/Rac1 Rho GTPase

**Figure 6:**
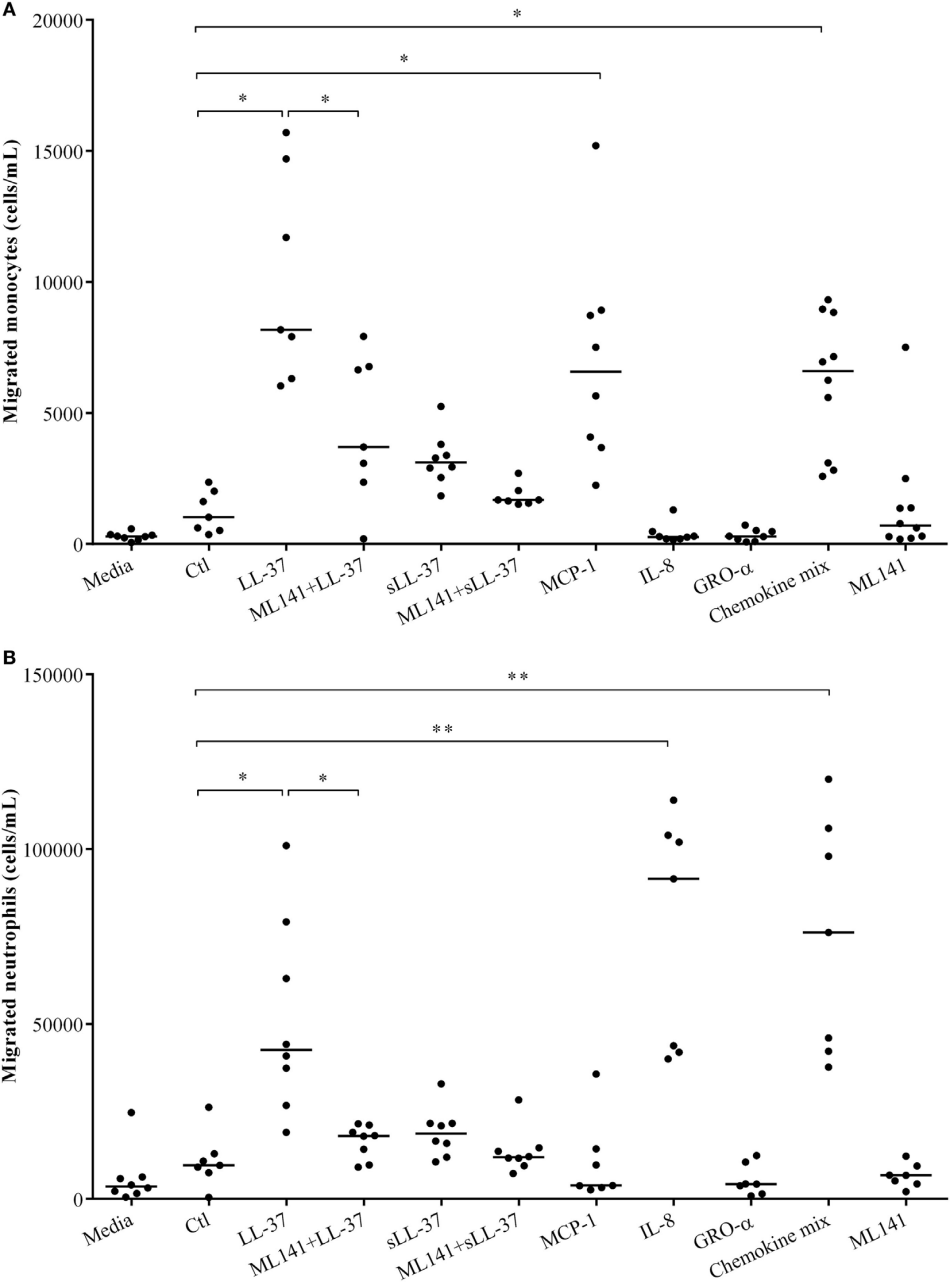
Cdc42/Rac1 Rho GTPases controls LL-37-mediated leukocyte migration. Human peripheral blood-derived mononuclear cells (PBMCs) were pre-incubated with the Cdc42/Rac1 inhibitor ML141 (10 µM) for 1 h prior to stimulation with either LL-37 or sLL-37 (5 µM each). PBMC tissue culture supernatants collected after 24 h were used in transwell cell migration assays to monitor the migration of **(A)** THP-1 monocytic cells and **(B)** human neutrophils. RPMI medium spiked with chemokines monocyte chemotactic protein-1, IL-8, and GRO-α (30 ng/ml each) were used as experimental controls as indicated. Results shown are from seven independent experiments using PBMCs isolated from independent donors (*n*-7). Each dot represents an independent donor and the median line is shown for each condition. Kruskal–Wallis analysis of variance with *Dunn’s* multiple comparison test was used for statistical analyses (*p < 0.05, **p < 0.005).

We have demonstrated that LL-37-induced production of chemokine IL-8, which recruits neutrophils is dependent on Cdc42/Rac1 Rho GTPase in macrophage-like THP-1 cells (Figure 1), in human PBMC (Figure 4) and MDM (Figure 5). LL-37 is also known to induce the production of chemokines such as MCP-1 that recruit monocytic cells (10). Similarly, we demonstrated production of MCP-1 following LL-37 stimulation in PBMC (Figure 4C). Therefore, in this study, we further examined LL-37-mediated recruitment of human monocytes and neutrophils, in the presence and absence of the inhibitor ML141, in cell migration assays. Human PBMCs were stimulated with peptides, either LL-37 or sLL-37, in the presence and absence of the inhibitor ML141. TC supernatants obtained from stimulated human PBMC were used in cell migration assays. Human recombinant chemokines MCP-1, IL-8, and GRO-α were used as controls in these assays. The monocyte chemoattractant MCP-1 significantly increased the migration of THP-1 monocytic cells, whereas the neutrophil chemokine IL-8 did not (Figure 6A). In contrast, the neutrophil chemoattractant IL-8 significantly increased the migration of human neutrophils, but MCP-1 did not (Figure 6B). GRO-α alone did not increase the migration of either monocytes or neutrophils. It is known that GRO-α recruits neutrophils *via* receptor CXCR2 independent of CXCR1, whereas IL-8 engages both these receptors (30, 31). Moreover, the affinity of CXCR2 was shown to be higher for IL-8 compared to GRO-α (32). Based on these studies, it is possible that the concentration (30 ng/ml) used for recombinant chemokines may not be sufficient for GRO-α-mediated neutrophil migration in this assay. Nevertheless, GRO-α was used as a negative control in this assay. A chemokine mix (MCP-1, IL-8, and GRO-α) was used as an overall positive control as it increased the migration of both monocytes and neutrophils (Figure 6). These results confirmed the specificity of the recombinant chemokines used as controls in the cell migration assays. We demonstrated that LL-37-induced monocyte cell migration was inhibited by ~50% (Figure 6A) and that of human neutrophils by ~70% (Figure 6B), in response to TC supernatants obtained from PBMC pretreated with the inhibitor ML141. These results functionally confirmed that the Cdc42/Rac1 Rho GTPase pathway plays a key role in controlling LL-37-mediated recruitment of leukocytes such as monocytes and neutrophils.

### Cdc42 GTPase Is Activated in Response to LL-37

**Figure 7:**
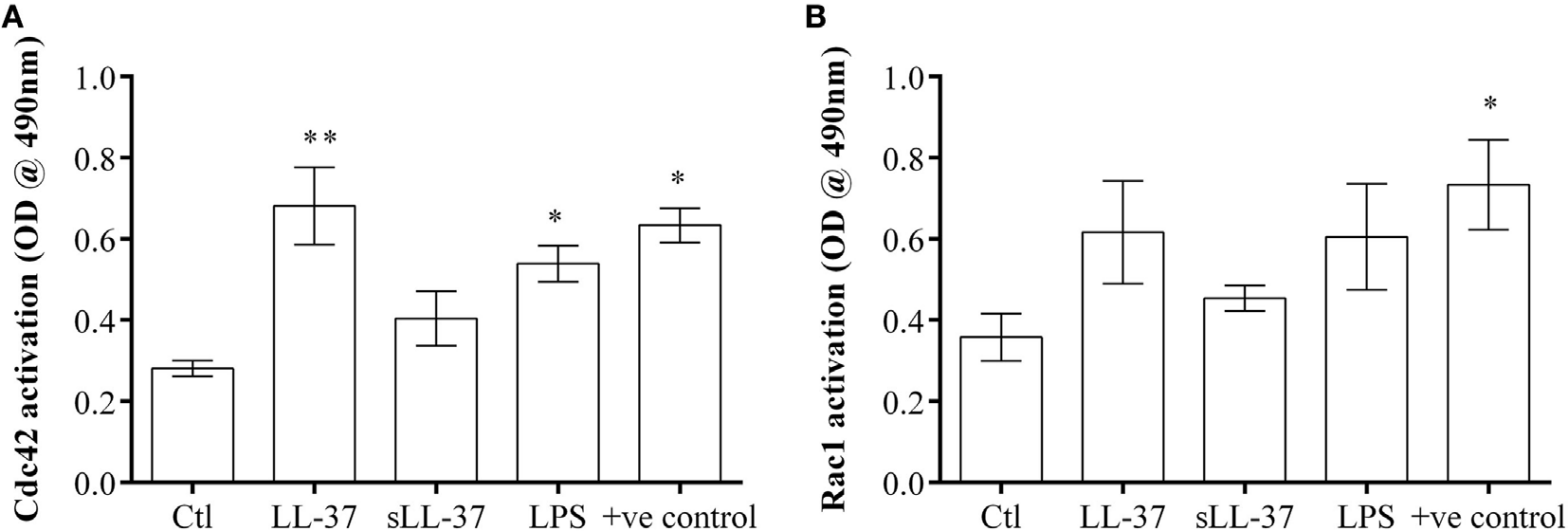
LL-37 activates Cdc42 Rho GTPase. Macrophage-like THP-1 cells were serum starved for 1 h prior to stimulation with either LL-37 (5 µM), sLL-37 (5 µM each), or lipopolysaccharide (10 ng/ml), and cell lysates collected after 5 min. The activities of **(A)** Cdc42 and **(B)** Rac1 Rho GTPases were determined using respective G-LISA assays. Positive control refers to active Cdc42 or Rac 1 protein provided in the G-LISA kit by the manufacturer. Results shown as mean ± SE of six independent experiments (*n* = 6). Analysis of variance with Bonferroni’s *post hoc* test was used for statistical analyses (*p < 0.05, **p < 0.005).

We demonstrated that the Cdc42/Rac1 pharmacological inhibitor ML141 significantly suppressed production of LL-37-induced chemokines in macrophage-like THP-1 cells (Figure 1), human PBMC (Figure 4), and MDM (Figure 5). We also showed that the inhibitor significantly suppressed LL-37-mediated JNK MAPK phosphorylation in both macrophage-like THP-1 cells (Figure 3) and in primary human MDM (Figure 5D). These results are consistent with previous studies demonstrating that LL-37-induced responses and signaling mechanisms are similar in macrophage-like THP-1 cells and in primary human blood-derived cells such as PBMC and MDM (4, 10). Therefore, we performed further mechanistic studies in the macrophage-like THP-1 cells. As the pharmacological inhibitor ML141 suppressed LL-37-mediated migration of monocytes and neutrophils (Figure 6), this indicated that Cdc42/Rac1 activity may be involved in the peptide-mediated response. Therefore, we examined activation of Cdc42 and Rac1 independently in the presence and absence of LL-37. Macrophage-like THP-1 cells were stimulated with either LL-37 or sLL-37 for 5 min, and the activations of Cdc42 and Rac1 Rho GTPase were analyzed using the respective G-LISA assays. We showed that stimulation with LL-37 significantly increased the activation of Cdc42 GTPase (Figure 7A), whereas that of Rac1 was modest and not statistically significant (Figure 7B). To further delineate whether LL-37 acts as a GEF, or alters human Dbs-induced GEF activity, we performed a fluorophore-based GEF assay. We demonstrated that the peptides LL-37 or sLL-37 did not exhibit GEF activity to facilitate the exchange of GDP to GTP for the activation of Cdc42 GTPases (Figure S2 in Supplementary Material). Taken together, these results demonstrated that Cdc42 Rho GTPase is activated in response to LL-37, but the peptide itself does not facilitate subsequent GEF activity.

### Knockdown of Cdc42 Rho GTPase Suppresses LL-37-Induced Chemokine Production

**Figure 8:**
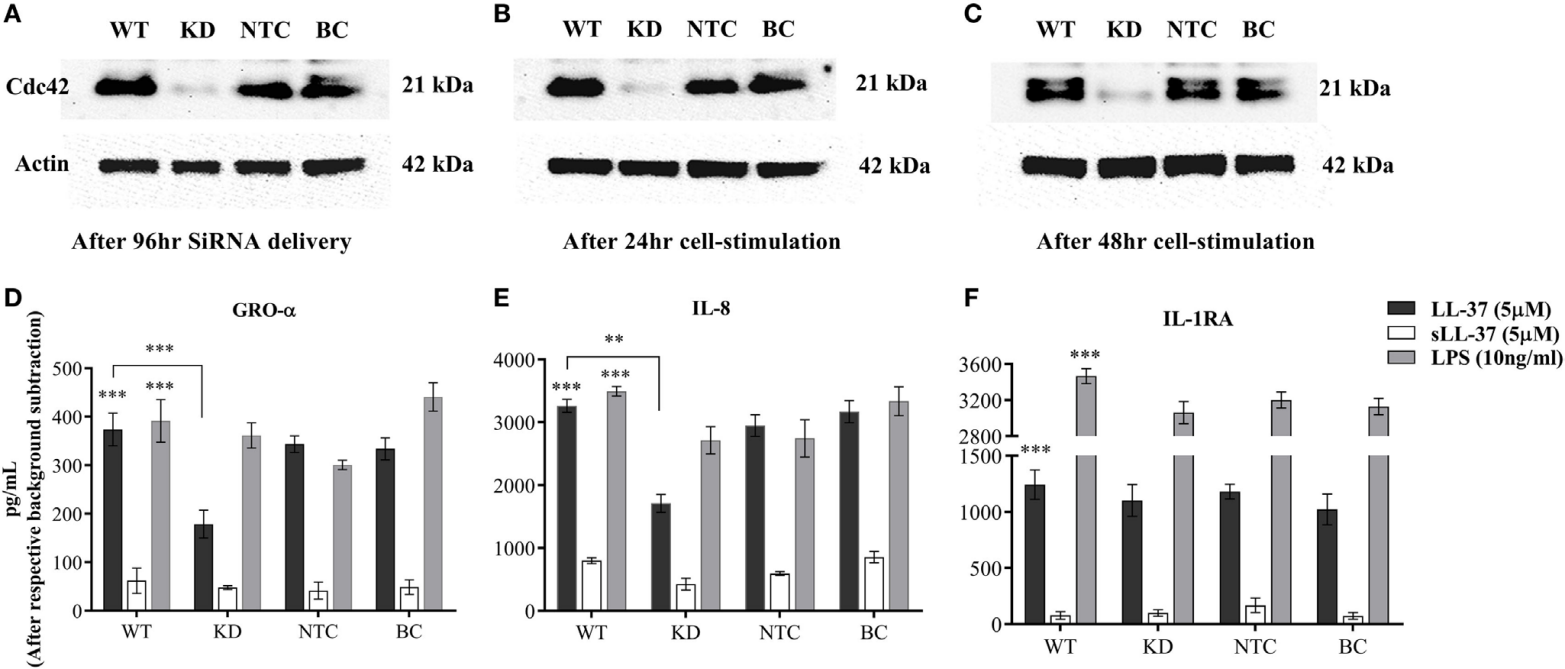
Knockdown of Cdc42 Rho GTPase suppresses LL-37-induced chemokine production, but not anti-inflammatory cytokine IL-1RA. Human monocytic THP-1 cells were treated with either human Cdc42 Accell SiRNA smartpool (1 µM) or non-target control (NTC) Accell SiRNA smartpool (1 µM) for 96 h. The efficiency of knockdown was determined by western blots probing with Cdc42 antibody and protein loading using actin antibody, after **(A)** 96 h of SiRNA delivery, and subsequently following cell differentiation to plastic-adherent, macrophage-like THP-1 cells after additional **(B)** 24 and **(C)** 48 h. Macrophage-like THP-1 cells, either wild type, knockdown (KD), or cells treated with 50 µl SiRNA buffer (BC), were stimulated with LL-37 (5 µM), sLL-37 (5 µM each) or lipopolysaccharide (10 ng/ml). Tissue culture supernatants were monitored by ELISA for the production of chemokines **(D)** GRO-α and **(E)** IL-8 after 24 h, and for **(F)** anti-inflammatory cytokine IL-1RA after 48 h. Results shown as mean ± SE of three independent experiments (*n* = 3). Analysis of variance with Bonferroni’s *post hoc* test was used for statistical analyses (**p < 0.005, ***p < 0.0005).

As Cdc42 Rho GTPase was activated in response to LL-37 (Figure 7A), we further monitored the effect of Cdc42 knockdown (KD) on LL-37-mediated responses. Cdc42 KD was confirmed using immunoblots after 96 h siRNA treatment (Figure 8A), and subsequently after additional 24 and 48 h (Figures 8B,C, respectively). This confirmed the knockdown of Cdc42 expression at the time point of cell stimulation and, at the time points, TC supernatants were collected for the assays. LDH abundance was monitored in TC supernatants to examine cytotoxicity, which showed that Cdc42 KD was not lethal to the cells after either 96 h of SiRNA delivery or subsequently after 24 or 48 h (Figure S3 in Supplementary Material). LL-37-induced chemokine production (GRO-α and IL-8) was significantly inhibited ~50% in the Cdc42 KD cells (Figures 8D,E). Whereas, LL-37-induced IL-1RA production was not suppressed in Cdc42 KD cells (Figure 8F). These results were similar to the results obtained using the pharmacological inhibitor ML141 (Figures 1, 2, 4, and 5). Taken together, our results suggest that the Cdc42 Rho GTPase pathway plays a key role in LL-37-mediated chemokine production.

### LL-37-Induced Cdc42 Rho GTPase Activity and Chemokine Production Is Mediated Through GPCR Signaling

**Figure 9:**
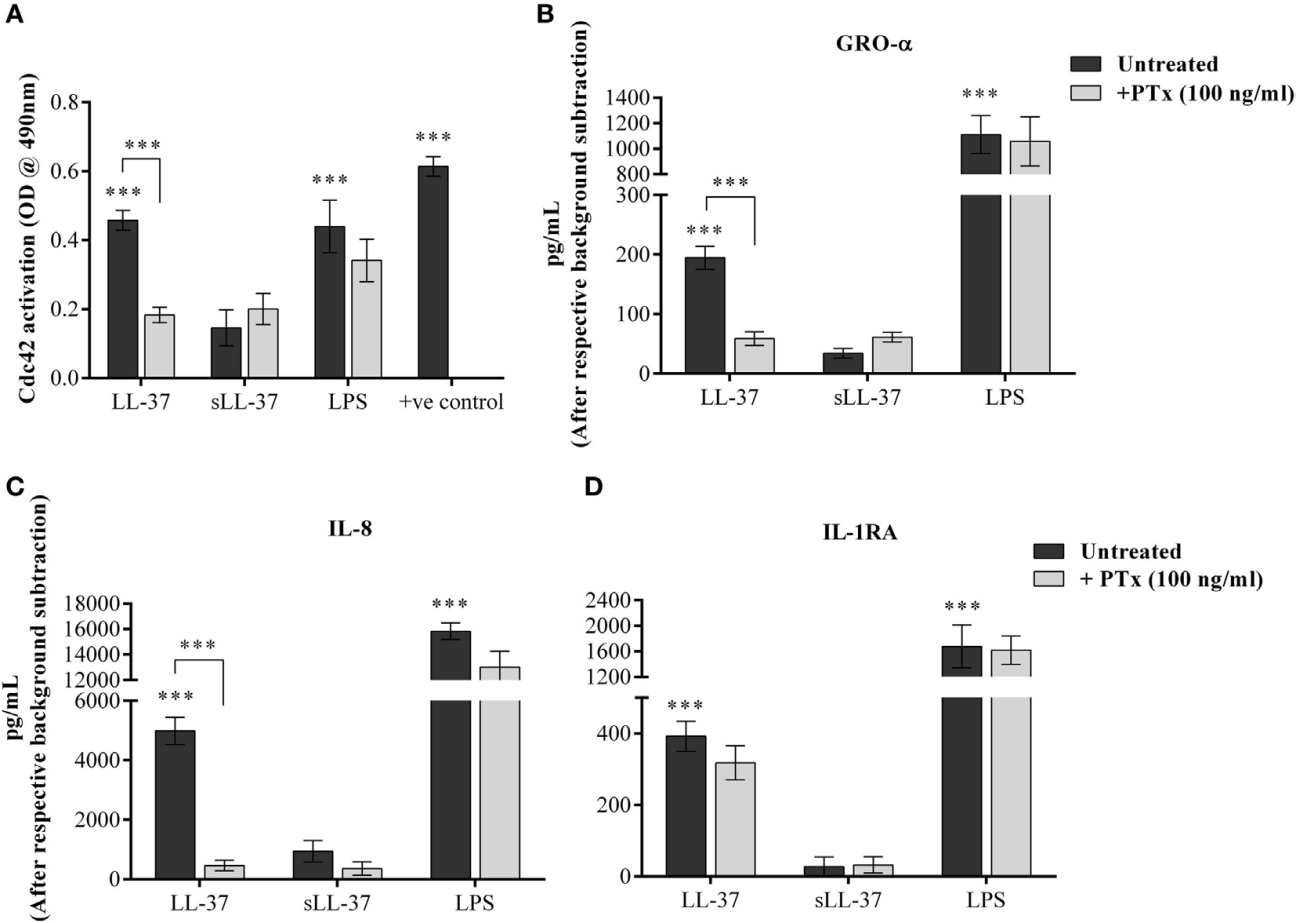
LL-37-induced activation of Cdc42/Rac1 Rho GTPase and chemokine secretion is mediated through G protein-coupled receptor (GPCR). Macrophage-like THP-1 cells were treated 100 ng/ml of the GPCR inhibitor pertussis toxin for 1 h, prior to stimulation with either LL-37 (5 µM), sLL-37 (5 µM each), or lipopolysaccharide (10 ng/ml). **(A)** Cells lysates were monitored for Cdc42 activation after 5 min of stimulation. Results shown as mean ± SE of five independent experiments (*n* = 5). Tissue culture supernatants were monitored by ELISA for the production of chemokines **(B)** GRO-α and **(C)** IL-8 after 24 h, and **(D)** anti-inflammatory cytokine IL-1RA after 48 h. ELISA results shown are mean ± SE of six independent experiments (*n* = 6). Analysis of variance with Bonferroni’s *post hoc* test was used for statistical analyses (***p < 0.0005).

Cdc42 is known to function in association with GPCR signaling, similar to other G-proteins (33). Therefore, we examined whether LL-37-induced activation of Cdc42 Rho GTPase and chemokine production is also mediated through GPCR pathway. We evaluated LL-37-induced responses in the presence of the GPCR inhibitor PTx. We showed that PTx significantly suppressed LL-37-mediated activation of Cdc42 Rho GTPase (Figure 9A). In addition, LL-37-induced production of chemokines GRO-α and IL-8 were significantly suppressed between 70 and 90%, in the presence of PTx (Figures 9B,C). In contrast, LL-37-induced IL-1RA production was not altered in the presence of PTx (Figure 9D). These results demonstrated that LL-37-mediated Cdc42 Rho GTPase activation and subsequent chemokine production involves GPCR signaling, but LL-37-induced production of the anti-inflammatory cytokine IL-1RA is not dependent on the GPCR-to-Cdc42 Rho GTPase pathway.

## Discussion

LL-37 is a multifunctional immunomodulatory peptide with opposing functions, acting both as an effector and regulator of inflammation (8, 9). LL-37 facilitates pro-inflammatory responses, primarily recruitment of leukocytes to the site of infections, which aids in the clearance of infections (34–36). LL-37 can function as a direct leukocyte chemoattractant, as well as indirectly promoting recruitment of leukocytes by inducing the production of chemokines (5, 35). Conversely, LL-37 can control the process of inflammation; LL-37 engages with intracellular receptors to alter TLR-to-NFκB signaling cascades in the presence of infectious challenge and suppresses endotoxin-induced inflammation (4, 8, 37). This duality of LL-37-mediated functions is also evident in the context of cytokine-induced inflammation; LL-37 can synergistically enhance inflammatory cytokines, e.g., IL-1β and GMCSF-induced responses (38), and conversely suppress cytokine IL-32-induced downstream inflammatory responses, in blood-derived monocytic cells (10). Consistent with this, LL-37 can induce the production of pro-inflammatory chemokines (e.g., IL-8 and GRO-α), as well as that of anti-inflammatory cytokines, e.g., IL-1RA (9, 10). Despite research in the last two decades clearly demonstrating both pro- and anti-inflammatory functions of LL-37, mechanisms that regulate the opposing functions of this peptide have not been completely elucidated.

Previous studies have suggested that LL-37 engages with different interacting protein partners or receptors, depending on the cellular microenvironment, to influence the activity of diverse signaling pathways (37, 39). Specific GPCRs have been demonstrated to be involved in some of the functions mediated by LL-37, which is primarily facilitated by MAPK signaling (11, 37, 40). GPCRs are known to engage Rho GTPases to activate downstream MAPK signaling pathways (12, 41, 42). Recent studies have demonstrated that similar to LL-37, Rho GTPases can also differentially modulate, i.e., both promote and control the process of inflammation (17, 18). Therefore, we hypothesized that Rho GTPases may be involved in the regulation of LL-37-mediated immune functions. In this study, we show that inhibition Cdc42/Rac1 Rho GTPase significantly suppress LL-37-induced secretion of chemokines. We further demonstrate that LL-37 induces the activation of Cdc42 Rho GTPase, and that the knockdown of Cdc42 suppresses LL-37-induced chemokine production. We also demonstrate that inhibiting Cdc42/Rac1 Rho GTPase suppresses LL-37-mediated leukocyte migration, in particular, migration of monocytes and neutrophils. It should be noted that LL-37 alone, at the concentration used, did not induce cell migration in our study (data not shown). These results suggest that the chemoattractant function of LL-37, likely that mediated indirectly by induction of chemokines subsequently facilitating the migration of leukocytes, is controlled by Rho GTPase namely Cdc42. This is corroborated by previous studies demonstrating that similar to LL-37, Rho GTPases also induce the release of chemokines such as MCP-1 and IL-8 (19, 43). Moreover, it is known that Rho GTPases promote cell migration and chemotaxis of leukocytes (8, 9, 44–46). However, as LL-37-induced chemokine production was not completely mitigated by either the Cdc42/Rac1 Rho GTPase pharmacological inhibitor or by Cdc42 knockdown, it is likely that there are alternate mechanisms that control the release of chemokines in response to LL-37. In contrast, we demonstrate that LL-37-induced production of the anti-inflammatory cytokine IL-1RA is not suppressed by either inhibition of Cdc42/Rac1 Rho GTPase or knockdown of Cdc42. This is the first study to provide direct evidence to demonstrate that LL-37-induced pro-inflammatory activity such as induction of chemokines and leukocyte migration, but not the ability of the peptide to mediate anti-inflammatory responses such as IL-1RA production, is selectively controlled by Cdc42 Rho GTPase activity. Even though we have only used IL-1RA for assessment of LL-37-mediated anti-inflammatory activity, this study opens up a new avenue to examine the role of Cdc42/Rac1 Rho GTPases (if any) in anti-inflammatory functions of LL-37. Moreover, as increased levels of LL-37 have been reported in chronic inflammatory diseases such as rheumatoid arthritis (47), psoriasis, and systemic lupus erythematosus (48, 49), results of this study imply that Cdc42 GTPase may be a useful drug target to selectively control LL-37-associated pro-inflammatory responses such as leukocyte recruitment to the site of inflammation, without compromising the anti-inflammatory functions of the peptide. Cell-specific deletion of Cdc42 GTPase has been shown to be effective in mitigating inflammation and plaque formation in atherosclerotic mice (18), thus providing the proof-of-concept that Cdc42 RhoGTPase may serve as a useful target for selectively regulating LL-37-mediated responses in chronic inflammatory diseases.

LL-37-mediated various functions are known to involve p38, JNK, and Erk1/2 MAPK in different cell types (10, 11, 40, 50). In this study, we demonstrate that control of LL-37-induced leukocyte recruitment by Cdc42/Rac1 Rho GTPases specifically involves the JNK MAPK signaling pathway. It has been previously demonstrated that Rho GTPases regulate chemokine expression *via* the JNK MAPK signaling pathway (17, 51), and that activation of Cdc42/Racl RhoGTPase is known to increase JNK phosphorylation (52). Overall, the JNK MAPK pathway is involved in chemokine production under various inflammatory conditions (53, 54). These studies support our findings that LL-37-mediated chemokine production and leukocyte recruitment involves the Cdc42/Rac1 Rho GTPase-JNK MAPK axis. However, it should be noted that there may exist some redundancy in MAPK signaling mechanisms in LL-37-induced chemoattractant activity, as previous studies have also demonstrated the involvement of p38 and Erk1/2 MAPK signaling in LL-37-induced chemokine, in particular, IL-8 production (55, 56). As LL-37-induced anti-inflammatory cytokine IL-1RA production was not dependent on either Rho or Ras GTPases, it is thus possible that the anti-inflammatory functions of LL-37 such as mitigation of endotoxin-induced inflammation involves the p38 and/or Erk1/2 MAPK signaling, but is independent of Rho GTPases. Indeed, previous studies have shown that the ability of LL-37 to suppress inflammatory responses involves the p38 and Erk1/2 signaling MAPK pathways (4, 10, 57, 58). Taken together, it can be surmised that the pro-inflammatory functions of LL-37 may be primarily controlled by Cdc42/Rac1 Rho GTPase pathway *via* JNK MAPK signaling, whereas the anti-inflammatory functions of the peptide may be independent of Rho GTPases selectively involving p38 and/or ERK1/2 MAPK signaling.

**Figure 10:**
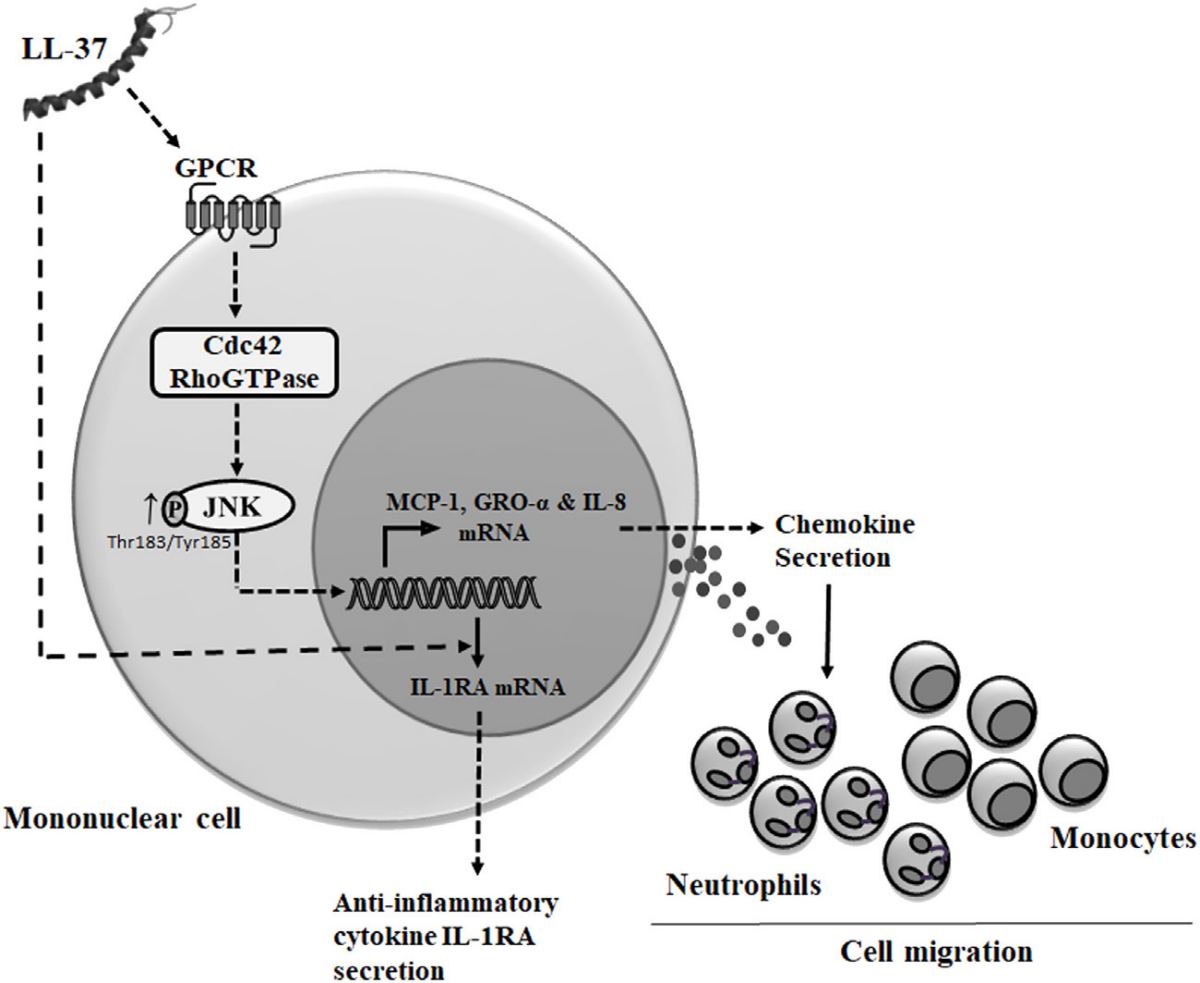
Model describing mechanism of LL-37-mediated chemokine secretion and leukocyte recruitment. Based on results from this study and previous studies, we propose that LL-37 can interact with G protein-coupled receptor (GPCR) to further activate (→) Cdc42 Rho GTPase (8, 11), resulting in enhanced phosphorylation of JNK mitogen-activated protein kinase (MAPK) (T183/Y185). The LL-37-GPCR-Cdc42 RhoGTPase axis signals *via* the JNK MAPK pathway to induce the production of chemokines and promote leukocyte migration. Alternatively, intracellular uptake of LL-37 may be mediated by other receptors (37) or using mechanisms similar to cell-penetrating peptides involving the cytoskeletal machinery (62). LL-37-induced anti-inflammatory cytokine IL-1RA production is independent of both GPCR and Cdc42/Rac1 Rho GTPases.

Cdc42 Rho GTPase is known to function in association with GPCR (33, 59). LL-37 has also been demonstrated to be an agonist for GPCRs such as fMet-Leu-Phe (fMLF), interaction of which results in increased cell migration (60). Consistent with this, we demonstrate that LL-37-induced chemokine secretion is mediated through the GPCR signaling. In contrast, we show that LL-37-induced IL-1RA production is not dependent on GPCR, which suggest that the anti-inflammatory functions of the peptide may be independent of GPCR signaling. This is consistent with our previous study demonstrating that the ability of LL-37 to suppress endotoxin-induced inflammatory responses are not inhibited by PTx and thus independent of GPCR (61). It is thus likely that the anti-inflammatory mechanisms of the peptide are mediated by the engagement with other receptors. We have previously demonstrated that LL-37 directly interacts with intracellular GAPDH to facilitate p38 MAPK signaling and downstream anti-inflammatory activity such as IL-10 production (37). However, the interaction of LL-37 with GAPDH also facilitates chemokine production (37), suggesting some redundancy in receptors and/or crosstalk between various pathways associated with immunomodulatory functions of LL-37, which needs to be fully delineated in future studies. Nevertheless, based on our overall findings, we submit the model that LL-37-GPCR interaction activates Cdc42 RhoGTPase subsequently engaging the JNK MAPK signaling pathway to induce chemokine production and downstream leukocyte recruitment (Figure 10), and that the mechanisms that underlie the ability of the peptide to control inflammation may be independent of this process. This is the first study to suggest that Cdc42 Rho GTPase activity may be the molecular toggle between LL-37-mediated inflammatory responses versus the anti-inflammatory functions of the peptide.

## Ethics Statement

Venous blood was collected from healthy volunteers with written informed consent, according to a protocol approved by the University of Manitoba Research Ethics Board.

## Author Contributions

NM and MH designed the study. MH primarily performed the experiments, analyzed the data, and wrote the manuscript. KGC performed the experiments with human PBMC and cell migration assays. NM conceived the study and extensively edited the manuscript. All the authors edited the manuscript.

## Conflict of Interest Statement

The authors declare that the research was conducted in the absence of any commercial or financial relationships that could be construed as a potential conflict of interest.
