# Supplementary material for: Host Defense Peptide LL-37-Mediated Chemoattractant Properties, but Not Anti-Inflammatory Cytokine IL-1RA Production, Is Selectively Controlled by Cdc42 Rho GTPase via G Protein-Coupled Receptors and JNK Mitogen-Activated Protein Kinase
Source: Front Immunol. 2018 Aug 13;9:1871. doi: 10.3389/fimmu.2018.01871 (PMC6104452; doi:10.3389/fimmu.2018.01871)

frontiers

Supplementary Information

Supplementary Figure 1:

Cdc42/Rac1 Rho GTPase does not control LL-37-mediated p38 and ERK1/2 MAPK phosphorylation. Macrophage-like THP-1 cells were pre-incubated with Cdc42/ Rac1 inhibitor ML141 (10 µM) for 1 h, prior to stimulation with either LL-37 or sLL-37 (5 µM each), or recombinant human IL-32γ (20 ng/ml) for 15 min. Cell lysates (each containing 20 µg total protein) were probed with either **(A)** phospho-p38 (T183/Y185) or **(B)** phospho-Erk1/2 antibodies in western blots. Antibodies against total-p38, total-ERK and β-actin were used as loading controls. Blots are representative of five independent experiments (n=5).

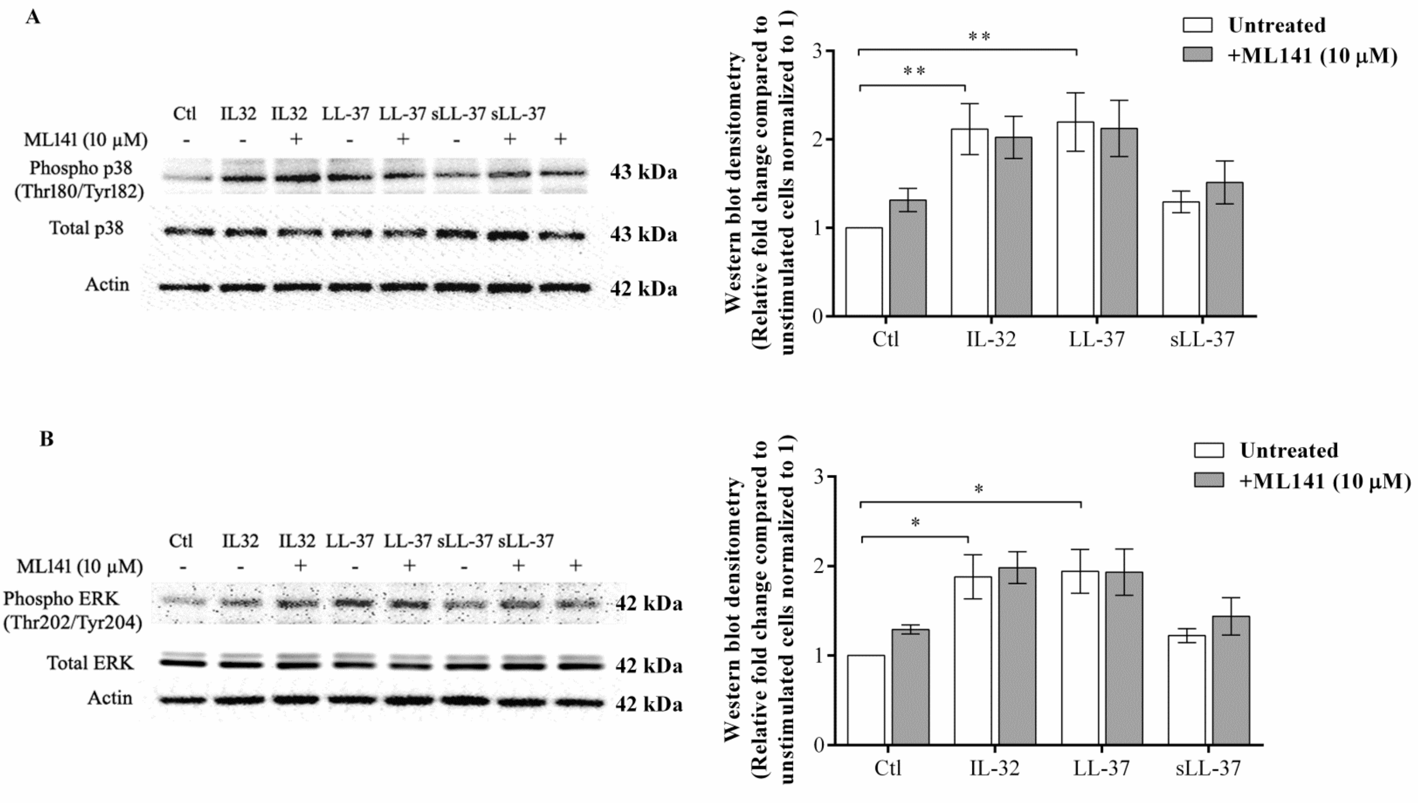

Supplementary Figure 2:

LL-37 does not act as a guanine nucleotide exchange factor, and does not alter human Dbs-induced GEF activity. The test samples were treated mixture containing Cdc42 Rho GTPases and fluorescent nucleotide analog N-methylanthraniloyl-GTP (mant-GTP), and the uptake of mant-GTP was measured using a fluorospectrometer at excitation 360 nm and emission 440 nm, every 30s for 60 min to record the sprectrum. Results shown as mean ± standard error of four independent experiments (n=4). ANOVA with Bonferroni's posthoc test was used for statistical analyses.

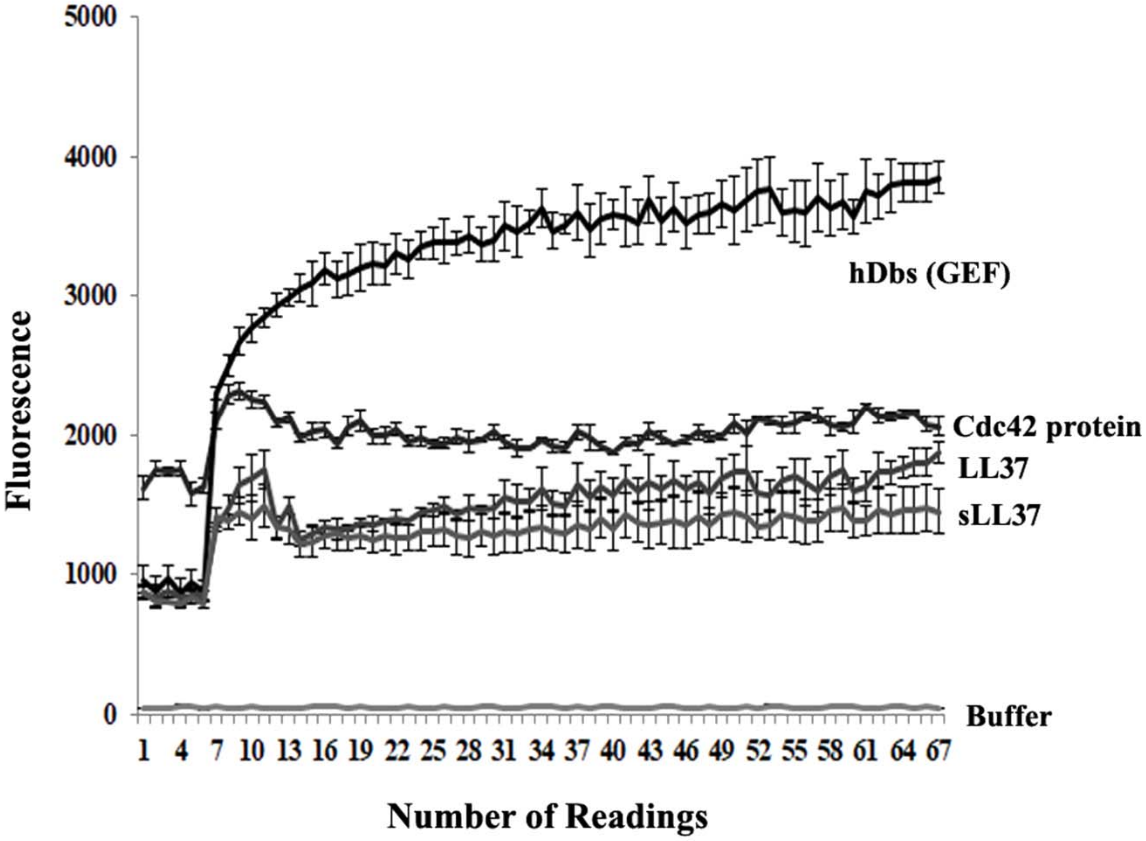

Supplementary Figure 3:

Knockdown of Cdc42 GTPase is not cytotoxic. Human monocytic THP-1 cells were treated with either human Cdc42 Accell SiRNA smartpool (1 μM) or non-target control (NTC) Accell SiRNA smartpool (1 μM) for 96 h. Wild type untreated cells (WT) or cells treated with 50 μL SiRNA buffer (BC) were used as paired controls. Plastic adherent macrophage-like THP-1 cells were stimulated with either LL-37 (5 μM), sLL-37 (5 μM each) or LPS (10 ng/ml), and tissue culture supernatants were monitored for the production of LDH by ELISA, after **(A)** 96 h of SiRNA delivery, and **(B)** 24 h and **(C)** 48 h after cell stimulation. Results shown as mean ± standard error of eight independent experiments (n=3).

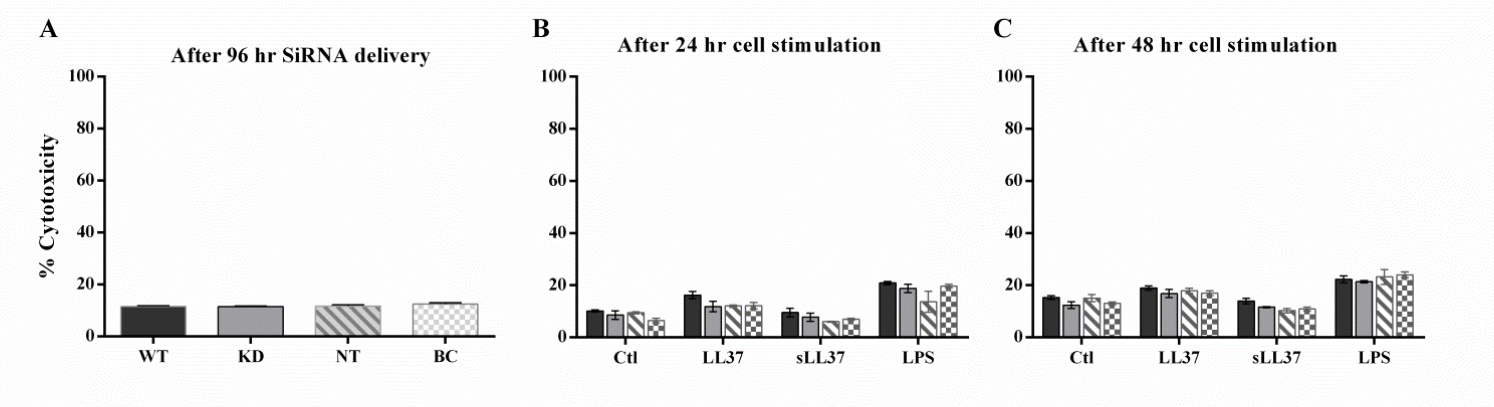

Supplement: Supplementary file 1 [file Data_Sheet_1.pdf]
